# Nicotine’s Effects on Schizophrenia-like Symptoms in a Mice Model: Time Matters

**DOI:** 10.3390/brainsci14090855

**Published:** 2024-08-25

**Authors:** Ana Carolina Dutra-Tavares, Luciana Araújo Couto, Thainá P. Souza, Anais Bandeira-Martins, Juliana Oliveira Silva, Claudio C. Filgueiras, Anderson Ribeiro-Carvalho, Alex C. Manhães, Yael Abreu-Villaça

**Affiliations:** 1 Departamento de Ciências Biomédicas e Saúde, Instituto de Biologia Roberto Alcantara Gomes, Universidade do Estado do Rio de Janeiro (UERJ), Cabo Frio 28905-320, RJ, Brazil; dutratavaresana@gmail.com; 2 Laboratório de Neurofisiologia, Departamento de Ciências Fisiológicas, Instituto de Biologia Roberto Alcantara Gomes, Universidade do Estado do Rio de Janeiro UERJ, Rio de Janeiro 20550-170, RJ, Brazilthainasouza2@gmail.com (T.P.S.); anais-band@hotmail.com (A.B.-M.); o.silvajuliana@yahoo.com.br (J.O.S.); ccfilg@yahoo.com.br (C.C.F.); yael.villaca@uerj.br (Y.A.-V.); 3 Departamento de Ciências, Faculdade de Formação de Professores, UERJ, São Gonçalo 24435-005, RJ, Brazil; ribeiro_carvalho@yahoo.com.br

**Keywords:** psychosis, positive symptoms, pre-pulse inhibition, tobacco, E-cigarette, smoking, comorbidity, NMDA receptor antagonism

## Abstract

Tobacco consumption in schizophrenia (SCHZ) patients is highly prevalent. Data support the occurrence of sequential events during comorbidity establishment, and both smoking first, SCHZ second and SCHZ first, smoking second sequences have been proposed. To investigate whether these two possibilities lead to distinct outcomes of comorbidity, we used a phencyclidine-induced SCHZ model and nicotine exposure as a surrogate of smoking. C57Bl/6 mice were submitted to a protocol that either began with 4 days of phencyclidine exposure or 4 days of nicotine exposure. This period was followed by 5 days of combined phencyclidine + nicotine exposure. Locomotor sensitization and pre-pulse inhibition (PPI) were assessed due to their well-known associations with SCHZ as opposed to rearing, an unrelated behavior. Nicotine priming potentiated phencyclidine-evoked sensitization. However, nicotine exposure after SCHZ modeling did not interfere with phencyclidine’s effects. In the PPI test, nicotine after SCHZ modeling worsened the phencyclidine-evoked deficiency in males. In contrast, nicotine priming had no effects. Regarding rearing, nicotine priming failed to interfere with phencyclidine-mediated inhibition. Similarly, phencyclidine priming did not modify nicotine-mediated inhibition. The present results indicate that the sequence, either SCHZ-first or nicotine-first, differentially impacts comorbidity outcomes, a finding that is relevant for the identification of mechanisms of nicotine interference in the neurobiology of SCHZ.

## 1. Introduction

Schizophrenia (SCHZ) is a mental disorder for which a diagnosis requires the identification of two or more of the following symptoms: delirium, hallucination, disorganized speech, disorganized behavior, and negative symptoms. Furthermore, at least one of the first three symptoms should be present, as well as one of the following: functional impairment impacting work, interpersonal relationships, or self-care [1].

There is a high prevalence of SCHZ and a tobacco misuse comorbidity. Tobacco smoking in SCHZ patients is 2–4 fold higher than in individuals without a mental illness, reaching 50–90% in this subpopulation [2,3,4]. These data corroborate evidence that, in spite of the overall smoking rates declining over the last few decades, the reduction in smoking in individuals with SCHZ has been significantly less clear [5,6,7]. Further aggravating the issue is that disproportionately high tobacco smoking rates in SCHZ patients are associated with heavier smoking patterns and more severe nicotine addiction than the measures from nonpatient smokers [8,9].

These disorders’ comorbidities are established early. Regular use and abuse of conventional tobacco products [10] and electronic nicotine delivery systems (ENDS) [11,12] characteristically begin during the transitional period between adolescence and adulthood. Similarly, this period is critical to SCHZ, as this is when its prodromal phase, characterized by the emergence of positive pre-psychotic symptoms as well as non-specific cognitive and negative symptoms, first manifest, and it is also when these symptoms escalate to a frank psychotic phase [13,14,15].

Even though these data lead to the impression of a strict temporal coincidence in the developmental events associated with these disorders’ initial stages, there are plenty of data supporting the notion that these mental health issues occur in sequence. In this regard, reports of a high prevalence of tobacco use (59–78%) already present in first-episode psychosis patients when compared with matched controls [16,17] point to a smoking first, SCHZ second sequence. In contrast, support for an SCHZ first, smoking second sequence includes the identification of attenuated SCHZ symptoms well before the classic period of smoking initiation [18,19,20] and of psycho-behavioral characteristics and neural circuit disturbances characteristic of SCHZ before the onset of the prodromal phase [19,21,22]. These data, together with reports of increasing smoking rates after SCHZ diagnosis [23], suggest that a subpopulation of patients resort to smoking after SCHZ symptoms initially manifest.

Smoking has been associated with an augmented risk of SCHZ, higher symptom severity, and poor SCHZ outcomes, but paradoxically, beneficial effects of smoking have been identified, and nicotinic agents may represent a strategy to remediate SCHZ symptoms [24,25]. We hypothesize that, at least in part, conflicting data associated with comorbid nicotine misuse reflect the timing of developmental events that lead to comorbidity. However, despite studies aiming to verify whether smoking begins before or after SCHZ [26], there are scant evaluations of the contribution of early versus late smoking initiation toward outcomes. When we expand the search to include animal models, designs that involve the use of nicotine or nicotinic agents after modeling SCHZ both during the perinatal period [27,28,29,30,31,32] or later in life (either at adolescence or adulthood) [33,34,35] have reported either null or aggravated SCHZ-like deleterious effects, as well as nicotine-induced normalization of behavioral and physiological endpoints. In addition, a higher sensitivity to the rewarding properties of nicotine has been identified [36,37], which has been proposed to contribute to comorbidity. As for the impact of nicotine pretreatment on SCHZ-modeled rodents, this was the focus of only a handful of studies. The data were not discussed in light of the priming effects of nicotine, but nicotine pretreatment was shown to alleviate MK-801-induced SCHZ-like symptoms and cognitive impairment in mice [38], possibly by enhancing NMDA receptors’ responses [39]. A summary of the relevant papers that used animal models of SCHZ and nicotine exposure is shown in Table S1.

Even though there have been studies that investigated SCHZ and smoking misuse comorbidity, direct experimental approaches in humans are challenging. In this regard, the use of animal models allows better control of potential confounding factors and has been shown to provide useful information. Considering (1) that investigation of the initial stages of SCHZ might offer insights into the pathophysiology of this disorder, and it has been suggested as an important window for early intervention [40,41], in addition to (2) recent evidence pointing to nicotine interference in SCHZ-like alterations already occurring during this window [42,43,44,45,46,47], here, we investigated the behavioral consequences of two possible developmental sequences of nicotine misuse and SCHZ in mice models of this comorbidity.

To accomplish this, we chose a phencyclidine-induced SCHZ model and nicotine exposure as a surrogate of smoking. Phencyclidine is a non-competitive NMDA receptor antagonist. Its repeated administration has been validated and is widely used as a model in SCHZ experimental research [48,49]. In addition, the conversion of SCHZ high-risk individuals to psychosis coincides with dysregulation of the glutamatergic system [50]. Nicotine, in turn, is the most important psychoactive component of tobacco smoking and ENDS, being responsible for a wide range of tobacco smoking effects, including dysregulation of the neurotransmitter systems [51,52,53] involved in SCHZ pathophysiology [54,55]. C57Bl/6 mice were submitted to a behavioral sensitization protocol that either began with 4 days of phencyclidine exposure (Experiment 1) or 4 days of nicotine exposure (Experiment 2). These were followed by an additional 4 days of combined phencyclidine + nicotine exposure. Finally, on the next day, the mice were again exposed to the drugs and submitted to a pre-pulse inhibition (PPI) test. Locomotor sensitization and PPI deficits were chosen based on their well-known association with SCHZ [30,56,57,58]. We also assessed rearing, an ethological behavior not directly related to SCHZ symptomatology, as a means to infer the specificity of the interference of nicotine in the phencyclidine model. With these experimental designs, we were able to test the hypothesis that different sequences of modeling SCHZ and nicotine misuse result in distinct comorbidity outcomes.

## 2. Materials and Methods

### 2.1. Subjects

All experiments were sanctioned by the Ethical Committee for Animal Research (protocol#: CEUA/033/2018) of the Universidade do Estado do Rio de Janeiro and aimed to reduce the number of animals used and avoid animal distress, in compliance with Brazilian Law # 11.794/2008. All mice were kept in an institutional vivarium and housed in groups of 2–5 at 21–22 °C on a 12 h light/dark cycle (lights on at 1:00 a.m.). Food and filtered water were available *ad libitum*. The C57BL/6 mice were derived from a colony maintained at the *Universidade Federal Fluminense* (Niteroi, Brazil) for over 60 generations. Considering NIH recommendations, both males and females were used [59].

### 2.2. Experimental Protocol

**Figure 1:**
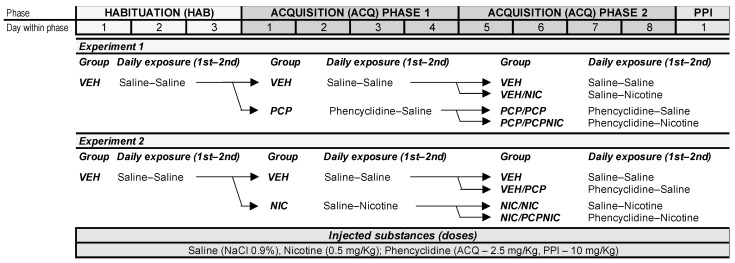
Timeline of experiments 1 and 2.

To fulfill its objective, this study was divided into 2 experiments. Experiment 1 was designed to mimic smoking onset after the expression of SCHZ-like behavioral characteristics, while Experiment 2 aimed to mimic smoking onset before the expression of SCHZ-like behavioral characteristics. Although each experiment addressed different aspects of the study, both used the same basic design for the assessment of behavioral sensitization in an open field (OF) arena and sensorimotor gating in a pre-pulse inhibition (PPI) test (Figure 1).

All OF and PPI testing sessions were carried out during the same photoperiod (lights on in the vivarium) in a sound-attenuated room illuminated by a 40 W fluorescent light (3 m high). Prior to testing, the animals were allowed to climatize and habituate to the test room for at least 10 min. Phencyclidine and nicotine exposure were chosen based on evidence that, in most cases, SCHZ symptoms manifest between late adolescence and early adulthood [14,60], which is the same period of tobacco and ENDS consumption establishment [61,62]. Previous studies showed phencyclidine-evoked locomotor sensitization as well as PPI and cognitive deficits at doses close to the ones used in the current study [42,63,64,65]. The dose of nicotine was chosen based on findings from our and other research groups indicating that it is not aversive and causes conditioned place preference [66,67]. Power analyses using G*Power software (version 3.1.9.6) and previous studies [42,46,68] using the SCHZ animal model used here were considered in determining the sample sizes per group.

#### 2.2.1. Behavioral Sensitization

The OF arena (Insight, São Paulo, Brazil) consisted of a transparent acrylic box (wall length of 46 cm and wall height of 43 cm). The arena was crisscrossed by two orthogonally positioned arrays of 16 parallel infrared beams each elevated 1.5 cm above the floor. Interruptions in the photocell beams, used in the measurement of the horizontal spontaneous locomotor activity (ambulation), were detected by a computer system, and the location of the animal was calculated by the software with a 0.1 s resolution. Ambulation was determined based on the traversed distance. In addition, all tests were recorded by a digital camera positioned 100 cm above the OF, and the video images were used to register vertical activity (number of rearings). After acclimatization to the test room, the mice received the daily injections and were individually placed in the center of the arena and allowed to explore for 40 min. The test apparatuses were cleaned with paper towels soaked in 35% ethanol and dried before each test.

- Habituation

The sensitization protocol in both Experiment 1 and Experiment 2 began with the habituation (HAB) phase, which was similar between experiments (Figure 1). C57BL/6 mice at early adulthood (60–70 days old at the beginning of the experiments) were handled by the experimenter and habituated to the subcutaneous (s.c.) injection cycles and OF tests. The mice received injections of a saline solution (NaCl 0.9%, 2.5 mL/Kg body mass) 10 min apart and were individually placed in the OF. These procedures were repeated once daily for 3 consecutive days (HAB1–3). After completing the HAB phase, the animals were assigned to either Experiment 1 or to Experiment 2, which began on the day immediately after HAB3.

- Experiment 1 (Exp. 1)

This experiment tested the effects of beginning the protocol by exposing mice to phencyclidine. During the first 4 days of the acquisition period (ACQ phase 1 (ACQ1–ACQ4); Figure 1), half of the animals assigned to this experiment continued to receive only saline injections (group VEH: ♀ = 20, ♂ = 21), while the other half were first injected with a phencyclidine solution (2.5 mg/Kg BM; Alomone Labs, Jerusalem, Israel) and with saline 10 min later (group PCP: ♀ = 24, ♂ = 26).

ACQ phase 2 (Figure 1) began the day after ACQ4 and lasted for 4 days (from ACQ5 to ACQ8). During this phase, the VEH and PCP groups were subdivided such that 4 groups were established, namely (1) group VEH (♀ = 10, ♂ = 10), composed of mice that received saline injections both during ACQ phase 1 and ACQ phase 2; (2) group VEH/NIC (♀ = 10, ♂ = 11), with mice that received saline injections during ACQ phase 1 and nicotine ditartrate (0.5 mg/Kg BM; Sigma Chemical Co. St. Louis, MO, USA) as their second daily injection during ACQ phase 2; (3) group PCP/PCP (♀ = 13, ♂ = 13), composed of mice that received phencyclidine as their first daily injection both during ACQ phase 1 and ACQ phase 2; and (4) group PCP/PCPNIC (♀ = 11, ♂ = 13), with mice that received phencyclidine as their first daily injection during ACQ phase 1 and that, in addition to phencyclidine, were exposed to nicotine as their second daily injection during ACQ phase 2.

- Experiment 2 (Exp. 2)

This experiment tested the effects of beginning the protocol by exposing mice to nicotine. As in Experiment 1, from ACQ1 to ACQ4, half of the animals assigned to this experiment continued to receive only saline injections (group VEH: ♀ = 20, ♂ = 21). However, the other half (group NIC: ♀ = 20, ♂ = 20) were first injected with saline and, 10 min later, with nicotine (Figure 1).

As in Experiment 1, ACQ phase 2 also began the day after ACQ4 and lasted for 4 days (from ACQ5 to ACQ8). During this phase (Figure 1), the VEH and NIC groups were subdivided such that 4 groups were established, namely (1) group VEH (♀ = 10, ♂ = 11), with mice that received saline injections during both ACQ phase 1 and ACQ phase 2; (2) group VEH/PCP (♀ = 10, ♂ = 10), with mice that which received saline injections during ACQ phase 1 and phencyclidine as their first daily injection during ACQ phase 2; (3) group NIC/NIC (♀ = 10, ♂ = 10), composed of mice that received nicotine as their second daily injection during both ACQ phase 1 and ACQ phase 2; and (4) group NIC/PCPNIC (♀ = 10, ♂ = 10), with mice that received nicotine as their second daily injection during ACQ phase 1 and that, during ACQ phase 2, in addition to nicotine, were exposed to phencyclidine as their second daily injection.

- Behavioral Assessment

The animals were tested in the OF immediately after the second injection in all phases of both experiments. For the analysis of locomotor activity, each 40-min session was divided into two consecutive 20 min intervals (Int1 and Int2), and ambulation was measured within each interval. The number of rearings was quantified on the first and last days of ACQ phase 1 (ACQ1 and ACQ4) and phase 2 (ACQ5 and ACQ8), and each session was divided into 10 min intervals. A trained observer blind to the group assignments registered the number of rearings in the first and last 10 min. The 3 HAB sessions (days) and corresponding subdivisions (intervals) allowed separate analyses of day-to-day and interval-to-interval behavioral alterations resulting from repeated testing. The experimental design of the ACQ phase, in addition to allowing day-to-day and interval-to-interval analyses, enabled investigation of the impacts of phencyclidine and nicotine separately, as well as their interactions as a function of the sequence of exposures.

#### 2.2.2. Sensorimotor Gating

A weak acoustic stimulus (pre-pulse) given before a strong startle-eliciting one (startle pulse) attenuates the response to the startle-eliciting stimulus in healthy individuals such that the allocation of attentional resources to salient environmental stimuli can be optimized [69]. This inhibiting effect of the pre-pulse, termed pre-pulse inhibition (PPI), is impaired in patients with SCHZ [69]. Deficits in PPI indicate disrupted sensorimotor gating and are suggested to cause sensory overload, thought disorder, and cognitive fragmentation in SCHZ patients [24].

The day after the last day of the behavioral sensitization protocol (ACQ8), the mice received their respective treatments and were immediately placed in the startle apparatus (Figure 1). In addition, to investigate whether neuroplastic events associated with the repeated exposures impacted the results, a separate group of mice was tested immediately after acute exposure to phencyclidine (PCPacute), phencyclidine and nicotine (PCPNICacute), or saline (VEHacute). The startle responses were measured as described previously (Panlab Startle and Fear Combined System; Harvard Apparatus, Holliston, MA, USA) [42,68]. Briefly, the test began with a 5-min acclimatization period in the startle apparatus under 65 dB of background white noise. Next, 10 startle pulse trials (120 dB, 40 ms) were generated. Then, 50 semi-randomized trials consisting of no pulse (0 dB), a startle pulse (P, 120 dB, 40 ms), or a pre-pulse (70 dB, 75 dB, or 80 dB) presented 100 ms before the startle pulse (pP) were presented. These were followed by 10 startle pulse trials (120 dB, 40 ms). A constant 20-s intertrial interval was applied throughout the test. The startle responses were measured every 1 ms in a 100-ms period following presentation of the startle stimulus. The startle response amplitudes were averaged separately for both types of trials (P and pP). The degree of pre-pulse inhibition is shown as the percentage of inhibition (%PPI), which was calculated according to the following formula: %PPI = 100 – (pP – P) × 100. Only the startle responses to the semi-randomized trials were used in this formula. Based on previous studies, we chose 10 mg/Kg as the dose of PCP to be administered before the PPI test [42,68,70]. The test apparatus was cleaned with diluted liquid soap and dried before each test.

### 2.3. Statistical Analysis

Data on the body mass, each phase of the sensitization protocols (HAB, ACQ phase 1, and ACQ phase 2), and PPI were first evaluated by global mixed-model analyses of variance (mxANOVAs) to reduce the likelihood of type 1 statistical errors that might have resulted from repeated testing. For the body mass, the Day was the within-subject factor. For the behavioral sensitization protocols, the within-subject factors were the Day and Interval, and for the PPI analysis, the dB intensity was the within-subject factor. Whenever applicable, Nicotine (exposed and non-exposed), Phencyclidine (exposed and non-exposed), and Sex were the between-subject factors. Lower-order mxANOVAs on each day or interval, univariate ANOVAs (uANOVAs), multivariate ANOVAs (mANOVAs), and Fisher’s protected least significant difference (FPLSD) and paired *t*-tests were used post hoc where appropriate. Specifically, for characterization of the development of behavioral sensitization, two criteria were used: a significant increase in activity in a given group when compared with the controls (as indicated by the FPLSD test) and a significant increase in activity throughout the ACQ period in a given group (as indicated by the paired *t*-test between pairs of ACQ days).

For the sake of simplicity, figures were segmented by sex only when significant sex interactions were observed. The sex’s effects in these cases were tested with unpaired *t*-tests between males and females of the same experimental group. Significance was assumed at the level of *p* < 0.05 for the main effects. However, for interactions at *p* < 0.1, we also examined whether lower-order main effects were detectable after subdivision of the interactive variables [71]. The criterion for the interaction terms was not used to assign significance to the effects but rather to identify interactive factors requiring subdivision for lower-order tests of the main effects [71]. All data were analyzed using the IBM SPSS Statistics for Windows, Version 24.0 (IBM Corp, Armonk, NY, USA) and compiled as means and standard errors.

## 3. Results

### 3.1. Body Mass

The body mass (Table S2) was not affected by exposure to phencyclidine, but repeated exposure to nicotine caused a subtle reduction specifically in Experiment 2 (Day: F_11,979_ = 16.0, *p* < 0.001; Day × Nicotine: F_11,979_ = 3.7, *p* < 0.001), reaching significance in ACQ4 (Nicotine: −3.1%, F_1,89_ = 5.6, *p* = 0.02) and remaining significant from then until the end of the experiment, irrespective of the introduction of phencyclidine (Nicotine, ACQ5: −2.9%, F_1,89_ = 4.1, *p* = 0.047; ACQ6: −2.8%, F_1,89_ = 3.9, *p* = 0.05; ACQ7: −3.2%, F_1,89_ = 5.2, *p* = 0.024; ACQ8: −3.3%, F_1,89_ = 5.5, *p* = 0.021; PPI: −3.4%, F_1,89_ = 5.3, *p* = 0.023). Sex failed to interact with either Nicotine or Phencyclidine.

### 3.2. Open Field: Locomotor Activity

#### 3.2.1. Habituation (Tables S3 and S4)

During this phase, all mice received saline injections and explored the OF. As expected, the mxANOVA showed both interday and intraday habituation. The longitudinal post hoc analysis showed that ambulation was highest in HAB1 in both experiments, and it decreased between intervals in all three HAB days. Even though a consistent reduction in ambulation during Int1 was observed throughout HAB, no reductions were evidenced between HAB2 and HAB3 during Int2, irrespective of the experiment. These data indicate that ambulation was stable by the end of HAB.

#### 3.2.2. Acquisition Phase 1 (Figure 2)

Experiment 1 allowed the investigation of changes in the time course of phencyclidine-evoked locomotion, while Experiment 2 was designed to investigate the impact of nicotine. The global mxANOVAs run for each experiment indicated that both phencyclidine and nicotine affected locomotion, albeit with distinct outcomes.

**Figure 2:**
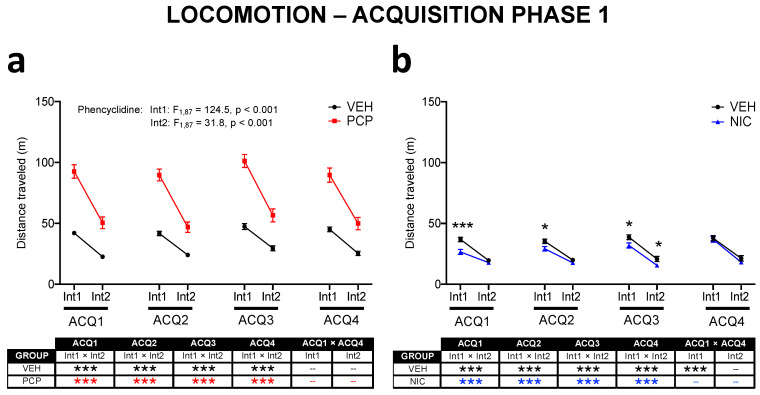
Distance traveled as a measure of locomotor activity in the open field during Acquisition (ACQ) phase 1 of Experiment 1 (**a**) and Experiment 2 (**b**). Each session (ACQ1, ACQ2, ACQ3, and ACQ4) lasted 40 min, which was divided into 2 intervals (Int1 and Int2) of 20 min each. The mice from Experiment 1 were either exposed daily to phencyclidine (PCP group) or received saline injections (VEH group). As for Experiment 2, the mice either received nicotine (NIC group) or saline (VEH group) before the tests. Values are means ± S.E.M. * *p* < 0.05, *** *p* < 0.001 vs. NIC group within a given interval.

The mice exposed to phencyclidine (Exp. 1, Figure 2a) were more active than the non-exposed ones (Phencyclidine: F_1,87_ = 78.1, *p* < 0.001), an effect that was evident during the whole of ACQ phase 1 (no Day × Phencyclidine interaction; no differences in ACQ1 × ACQ4 longitudinal analysis of PCP mice). Even though the hyperlocomotor effect was identified in both intervals (Int1, Phencyclidine: F_1,87_ = 124.5, *p* < 0.001; Int2, Phencyclidine: F_1,87_ = 31.8, *p* < 0.001), it was more evident during Int1, and the differences between the PCP and VEH mice faded throughout each session (Interval × Phencyclidine: F_1,87_ = 69.3, *p* < 0.001).

In contrast to the effect of phencyclidine, nicotine exposure (Exp. 2, Figure 2b) reduced locomotor activity (Nicotine: F_1,77_ = 4.1, *p* = 0.024; Day × Interval × Nicotine: F_3,231_ = 4.1, *p* = 0.007). The analysis of each day and interval showed a subtle hypolocomotor effect that was more evident in Int1. The lack of differences between the NIC and VEH mice on ACQ4 suggests attenuated effects of nicotine due to repeated exposure. This suggestion is corroborated by the longitudinal analysis, which showed that the locomotor activity of the NIC-exposed mice recovered throughout ACQ phase 1 (Int1: ACQ1 < ACQ4).

#### 3.2.3. Acquisition Phase 2 (Table 1, Figure 3)

ACQ phase 2 of Experiment 1 aimed to mimic smoking onset after the expression of SCHZ-related effects, as opposed to Experiment 2, which aimed to mimic smoking onset before the expression of SCHZ-related effects. The global mxANOVAs run for each experiment indicated that both phencyclidine and nicotine affected locomotion. It was also evident that the effects of these drugs were not consistent throughout the days and intervals of ACQ phase 2, and one drug interfered with the outcomes of the other (Table 1).

**Table 1:** Global analysis (mxANOVA) of locomotor activity from ACQ phase 2.

**Experiment 1: Effect or Interaction**	**F_d.f._, *p* Value**
Phencyclidine	F_1,83_ = 76.0, *p* < 0.001
Phencyclidine × Sex	F_1,83_ = 4.5, *p* = 0.036
Day × Phencyclidine	F_3,249_ = 2.1, *p* = 0.097
Interval × Phencyclidine	F_1,83_ = 340.8, *p* < 0.001
Interval × Nicotine	F_1,83_ = 4.0, *p* = 0.051
Interval × Phencyclidine × Nicotine	F_1,83_ = 3.1, *p* = 0.082
**Experiment 2: Effect or Interaction**	**F_d.f._, *p* Value**
Phencyclidine	F_1,73_ = 232.6, *p* < 0.001
Nicotine × Phencyclidine	F_1,73_ = 3.7, *p* = 0.058
Phencyclidine × Sex	F_1,73_ = 7.8, *p* = 0.006
Day × Interval × Phencyclidine	F_3,219_ = 2.7, *p* = 0.05
Day × Interval × Phencyclidine × Sex	F_3,219_ = 3.3, *p* = 0.021
Day × Interval × Nicotine × Phencyclidine × Sex	F_3,219_ = 2.8, *p* = 0.042
Interval × Phencyclidine	F_1,73_ = 49.1, *p* < 0.001

**Figure 3:**
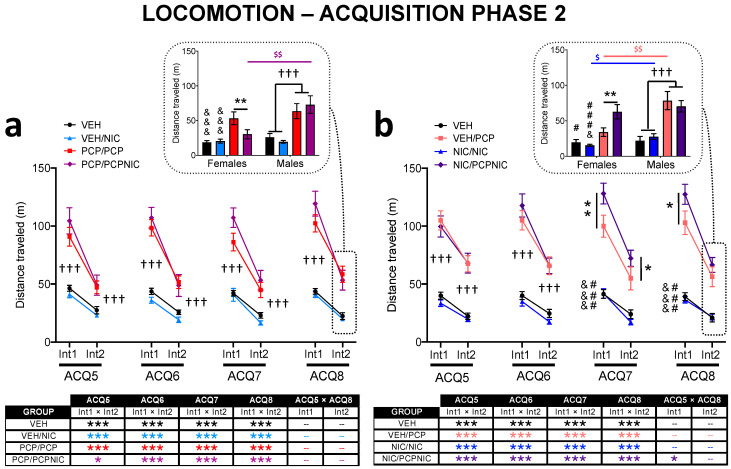
Distance traveled as a measure of locomotor activity in the open field during Acquisition (ACQ) phase 2 of Experiment 1 (**a**) and Experiment 2 (**b**). Each session (ACQ5, ACQ6, ACQ7, and ACQ8) lasted 40 min and was divided into 2 intervals (Int1 and Int2) of 20 min each. Data are shown separately for 4 experimental groups. The vehicle (VEH) group received saline injections throughout the experimental protocol. In Experiment 1, half of the mice that received saline injections in ACQ phase 1 received nicotine from ACQ5 to ACQ8 (VEH/NIC group). As for the mice that received phencyclidine during ACQ phase 1, half received 4 additional days of phencyclidine injections (PCP/PCP group), while the other half, in addition to phencyclidine, were exposed to nicotine (PCP/PCPNIC group). In Experiment 2, half of the mice that received saline injections in ACQ phase 1 received phencyclidine from ACQ5 to ACQ8 (VEH/PCP group). As for the mice that received nicotine during ACQ phase 1, half received 4 additional days of nicotine injections (NIC/NIC group), while the other half received both phencyclidine and nicotine (NIC/PCPNIC group). Values are means ± S.E.M. ^†††^ *p* < 0.001: for Experiment 1 (a), VEH and VEH/NIC vs. PCP/PCP PCP/PCPNIC, and for Experiment 2 (b), VEH and NIC/NIC vs. VEH/PCP and NIC/PCPNIC. * *p* < 0.05, ** *p* < 0.01 and *** *p* < 0.001 for comparisons between groups as indicated in the graphs or between intervals (boxes); ^&^ *p* < 0.05, and ^&&&^ *p* < 0.001 vs. PCP/PCP or VEH/PCP, while ^###^ *p* < 0.001 vs. NIC/PCPNIC and ^$^ *p* < 0.05 and ^$$^ *p* < 0.01 sex differences within a given group; different colors indicate the groups that were compared.

In Experiment 1 (Figure 3a), the pattern of the results was mostly similar to that identified during ACQ phase 1; the lower order mANOVAs identified phencyclidine-evoked increased locomotion at all analyzed days (ACQ5–ACQ8, PCP-exposed > VEH) and, while significant at both intervals, more robust at Int1. The longitudinal analysis of Int1 further indicated that phencyclidine exposure evoked a progressive increase in locomotor activity throughout ACQ; significant differences were observed between ACQ1 and ACQ8 (t_49_ = −2.6, *p* = 0.012) as well as between ACQ5 and ACQ8 (t_49_ = −2.2, *p* = 0.03), a pattern consistent with the development of locomotor sensitization. However, when the PCP/PCP and PCP/PCPNIC groups were analyzed separately, the longitudinal effects failed to reach significance (Figure 3a). Consistent with this latter finding, nicotine exposure did not affect phencyclidine-evoked hyperlocomotion at Int1 (ACQ5, ACQ6, ACQ7, and ACQ8: PCP/PCP = PCP/PCPNIC). Its sole impact was at Int2, when this drug had a punctual effect, mitigating the hyperlocomotor effect of phencyclidine at ACQ8 (Phencyclidine × Nicotine × Sex: F_1,83_ = 3.0, *p* = 0.087) in females (Figure 3a indent).

In Experiment 2 (Figure 3b), as expected, the introduction of phencyclidine had a major impact on locomotion. The increase in activity was significant for all analyzed ACQ days (ACQ5–ACQ8, PCP-exposed > VEH) and consistently more robust at Int1. Nicotine priming impacted phencyclidine-evoked locomotor sensitization. Its repeated exposure potentiated the hyperlocomotor effect of phencyclidine at ACQ7 Int1 (Nicotine × Phencyclidine: F_1,73_ = 4.1, *p* = 0.047) and Int2 (Nicotine × Phencyclidine: F_1,73_ = 3.8, *p* = 0.055) and at ACQ8 Int1 (Nicotine × Phencyclidine: F_1,73_ = 4.0, *p* = 0.049) in both males and females (Figure 3B). At Int2 of ACQ8, the potentiation evoked by nicotine was also evident (Nicotine × Phencyclidine × Sex: F_1,73_ = 5.0, *p* = 0.029), but restricted to females (Nicotine × Phencyclidine: F_1,36_ = 6.8, *p* = 0.013) (Figure 3B indent). The longitudinal analysis showed that the activity of the VEH/PCP mice was stable throughout ACQ phase 2, suggesting that more days of exposure to phencyclidine are necessary to cause a day-to-day increase in activity. Distinctively, consistent with the interference of nicotine in phencyclidine’s locomotor stimulatory effects, the activity of the NIC/PCPNIC mice progressively increased (Int1, ACQ5 × ACQ8), indicative of the development of locomotor sensitization (Figure 3b).

### 3.3. Open Field: Rearing

#### 3.3.1. Acquisition Phase 1 (Figure 4a,b)

The global mxANOVAs run for each experiment indicated that the number of rearings varied from session to session and also throughout each session, and both phencyclidine (Exp. 1, Phencyclidine: F_1,78_ = 65.3, *p* < 0.001; Day × Phencyclidine: F_1,78_ = 3.5, *p* = 0.064; Interval × Phencyclidine: F_1,78_ = 267.5, *p* < 0.001) and nicotine (Exp. 2, Nicotine: F_1,77_ = 413.1, *p* < 0.001; Day × Nicotine: F_1,77_ = 11.3, *p* = 0.001; Interval × Nicotine: F_1,77_ = 1188.9, *p* < 0.001) affected the results.

**Figure 4:**
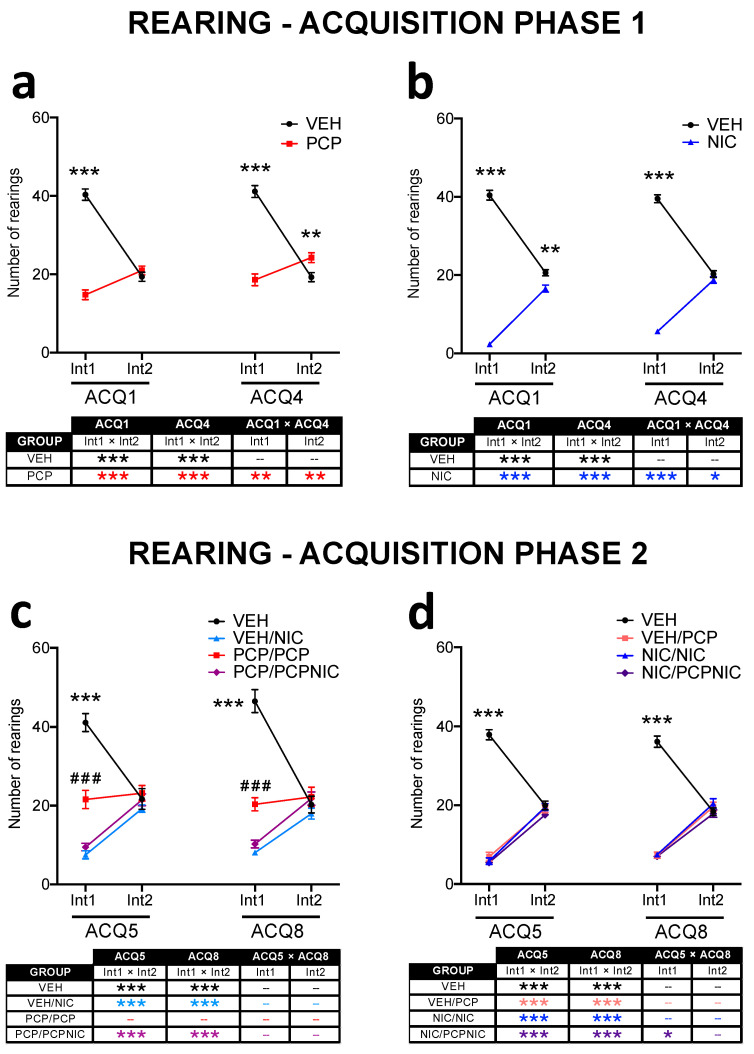
Number of rearings in the open field during Acquisition (ACQ) phases 1 (A and B) and 2 (C and D). The first and last 10 min (Int1 and Int2) of ACQ1, ACQ4, ACQ5, and ACQ8 were analyzed. During ACQ phase 1 of Experiment 1 (**a**), the mice were either exposed daily to phencyclidine (PCP group) or received saline injections (VEH group) from ACQ1 to ACQ4. As for ACQ phase 1 of Experiment 2 (**b**), the mice either received nicotine (NIC group) or saline (VEH group). During ACQ phase 2 of Experiment 1 (**c**), half of the mice that received saline injections in ACQ phase 1 received additional saline injections (VEH group), while the other half received nicotine from ACQ5 to ACQ8 (VEH/NIC group). As for the mice that received phencyclidine during ACQ phase 1, half received 4 additional days of phencyclidine injections (PCP/PCP group), while the other half, in addition to phencyclidine, were exposed to nicotine (PCP/PCPNIC group). During ACQ phase 2 of Experiment 2 (**d**), half of the mice that received saline injections in ACQ phase 1 received additional daily saline injections (VEH group), while the other half received phencyclidine (VEH/PCP group). As for the mice that received nicotine during ACQ phase 1, half received 4 additional days of nicotine injections (NIC/NIC group), while the other half received both phencyclidine and nicotine (NIC/PCPNIC group). Values are means ± S.E.M. * *p* < 0.05, ** *p* < 0.01 and *** *p* < 0.001 VEH vs. all other groups or for comparisons between intervals (boxes). ^###^ *p* < 0.001 PCP/PCP vs. all other groups.

Phencyclidine (Exp. 1, Figure 4a) and nicotine (Exp. 2, Figure 4b) transiently reduced the number of rearings at the beginning of each testing session, with effects evident at both ACQ1 and ACQ4. The differences between groups faded throughout each session in part due to expected reductions in this exploratory behavior in VEH mice but also due to a restoration in the number of rearings within each session and between sessions from ACQ1 to ACQ4 in the mice exposed to phencyclidine and nicotine.

#### 3.3.2. Acquisition Phase 2 (Table 2, Figure 4c,d)

The global mxANOVAs run for each experiment indicated that both phencyclidine and nicotine affected the number of rearings, the effects of these drugs were not consistent throughout the ACQ intervals, and one drug interfered with the outcomes of the other (Table 2).

**Table 2:** Global analysis (mxANOVA) of the number of rearings at ACQ phase 2.

**Experiment 1: Effect or Interaction**	**F_d.f._, *p* Value**
Phencyclidine	F_1,74_ = 9.0, *p* = 0.004
Nicotine	F_1,74_ = 86.3, *p* < 0.001
Phencyclidine × Nicotine	F_1,74_ = 23.4, *p* < 0.001
Day × Interval × Phencyclidine	F_1,74_ = 3.3, *p* = 0.072
Interval × Phencyclidine	F_1,74_ = 74.9, *p* < 0.001
Interval × Nicotine	F_1,74_ = 214.9, *p* < 0.001
Interval × Phencyclidine × Nicotine	F_1,74_ = 73.6, *p* < 0.001
**Experiment 2: Effect or Interaction**	**F_d.f._, *p* Value**
Nicotine	F_1,73_ = 158.4, *p* < 0.001
Phencyclidine	F_1,73_ = 160.9, *p* < 0.001
Nicotine × Phencyclidine	F_1,73_ = 112.2, *p* < 0.001
Day × Nicotine	F_1,73_ = 3.0, *p* = 0.087
Interval × Nicotine	F_1,73_ = 191.8, *p* < 0.001
Interval × Phencyclidine	F_1,73_ = 164.5, *p* < 0.001
Interval × Nicotine × Phencyclidine	F_1,73_ = 211.1, *p* < 0.001

During ACQ phase 2 of both experiments, as in phase 1, phencyclidine and nicotine transiently reduced the number of rearings at the beginning of each testing session. The reductions were evident at both ACQ5 (Exp. 1, Phencyclidine: F_1,74_ = 25.3, *p* < 0.001; Nicotine: F_1,74_ = 165.0, *p* < 0.001; Phencyclidine × Nicotine: F_1,74_ = 36.9, *p* < 0.001; Exp. 2, Nicotine: F_1,73_ = 266.8, *p* < 0.001; Phencyclidine: F_1,73_ = 231.6, *p* < 0.001; Phencyclidine × Nicotine: F_1,73_ = 221.4, *p* < 0.001) and ACQ8 (Exp. 1, Phencyclidine: F_1,74_ = 45.8, *p* < 0.001; Nicotine: F_1,74_ = 183.8, *p* < 0.001; Phencyclidine × Nicotine: F_1,74_ = 63.2, *p* < 0.001; Exp. 2, Nicotine: F_1,73_ = 214.1, *p* < 0.001; Phencyclidine: F_1,73_ = 218.6, *p* < 0.001; Phencyclidine × Nicotine: F_1,73_ = 205.4, *p* < 0.001).

In Experiment 1 (Figure 4c), while the mice primed with phencyclidine (PCP/PCP group) had higher rearing numbers than those exposed to nicotine alone (VEH/NIC) or to phencyclidine and nicotine (PCP/PCPNIC), exposure to nicotine alone or to phencyclidine and nicotine resulted in similar rearing numbers (VEH/NIC = PCP/PCPNIC), which is consistent with a predominant nicotine effect. The differences between groups faded throughout each session in part due to reductions in this exploratory behavior in VEH mice but also due to a restoration in the number of rearings in the mice exposed to phencyclidine and nicotine. In Experiment 2 (Figure 4d), the magnitude of the reduction identified at Int1 was equivalent to those in response to nicotine (NIC/NIC group), phencyclidine (VEH/PCP group), and co-exposure (NIC/PCPNIC).

### 3.4. Prepulse Inhibition

The global mxANOVA (Table 3) revealed that when phencyclidine preceded nicotine in the experimental protocol (Exp. 1), phencyclidine, nicotine, and sex affected the %PPI and the effects varied as a function of the pP dB intensity. When nicotine preceded phencyclidine (Exp. 2), the pP dB intensity and phencyclidine were again determinant of the results, but neither nicotine nor sex affected the %PPI.

**Figure 5:**
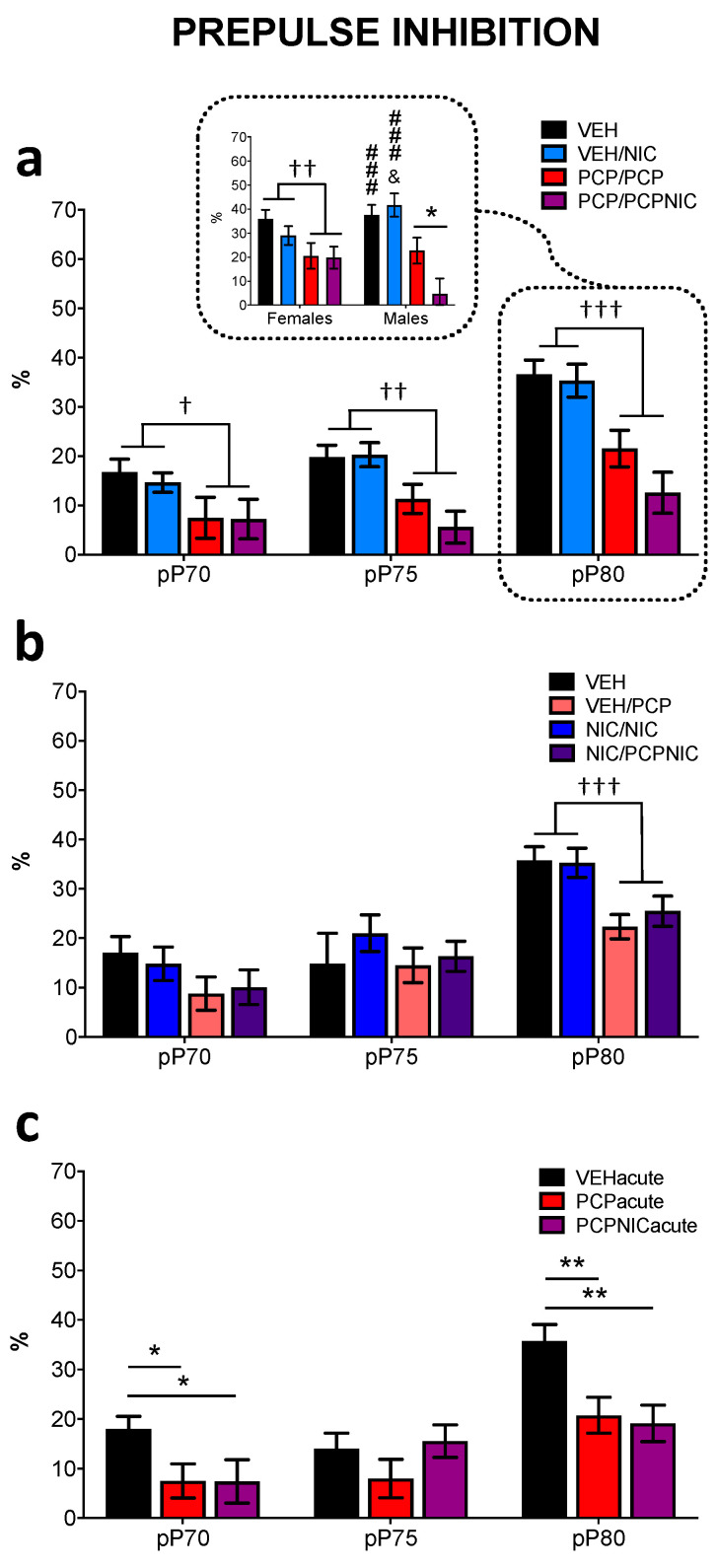
Percentage of pre-pulse inhibition (%PPI) at 70, 75, and 80 dB in males and females. For both Experiment 1 (**a**) and Experiment 2 (**b**), data are shown separately for 4 experimental groups. In the Experiment 1 indent, data from 80 dB pP are exhibited separately for males and females to show the interference of nicotine in phencyclidine-evoked deficits. The vehicle (VEH) group received saline injections throughout the experimental protocol. In Experiment 1 (**a**), half of the mice that received saline injections in ACQ phase 1 received nicotine from ACQ5 to PPI (VEH/NIC group). As for the mice that received phencyclidine during ACQ phase 1, half received 5 additional days of phencyclidine injections (PCP/PCP group), while the other half, in addition to phencyclidine, was exposed to nicotine (PCP/PCPNIC group). In Experiment 2 (**b**), half of the mice that received saline injections in ACQ phase 1 received phencyclidine from ACQ5 to PPI (VEH/PCP group). As for mice that received nicotine during ACQ phase 1, half received 5 additional days of nicotine injections (NIC/NIC group), while the other half received both phencyclidine and nicotine (NIC/PCPNIC group). In a separate experiment (**c**), the mice were tested immediately after acute exposures to phencyclidine and nicotine. VEHacute = vehicle group; PCPacute = acutely exposed to phencyclidine; PCPNIC = acutely exposed to phencyclidine and nicotine. ^†^ *p* < 0.05, ^††^ *p*< 0.01, and ^†††^ *p* < 0.001 for Experiment 1 (**a**), VEH and VEH/NIC vs. PCP/PCP PCP/PCPNIC, and for Experiment 2 (**b**), VEH and NIC/NIC vs.VEH/PCP and NIC/PCPNIC; ^&^ *p* < 0.05 vs. PCP/PCP, and ^###^ *p* < 0.001 vs. PCP/PCPNIC. * *p* < 0.05, ** *p* < 0.01.

Subsequent uANOVAs for each pP confirmed that phencyclidine had a major impact on the %PPI. In Experiment 2 (Figure 5b), the %PPI reduction reached significance when the P was preceded by the 80 dB pre-pulse (F_1,80_ = 16.6, *p* < 0.001). Distinctively, a more prolonged phencyclidine exposure in Experiment 1 (Figure 5a) reduced the %PPI even with lower pre-pulse levels (pP70: F_1,69_ = 6.9, *p* = 0.011; pP75: F_1,69_ = 19.6, *p* < 0.001; pP80: F_1,69_ = 30.3, *p* < 0.001). Another distinction between experiments was that in Experiment 1, when the P was preceded by the 80 dB pre-pulse, the %PPI reduction was sex-dependent and biased by nicotine exposure (Phencyclidine × Nicotine × Sex: F_1,69_ = 4.2, *p* = 0.044) such that in the PCP/PCPNIC males (Figure 5a indent), the PPI evoked by phencyclidine was further inhibited by nicotine exposure (PCP/PCPNIC < PCP/PCP).

The aforementioned results suggest that not only the order of the exposures but also their duration impacted the results. Accordingly, we then investigated whether the %PPI deficits would be identified after an acute, one-day exposure. The acute exposure reduced the %PPI (F_2,51_ = 4.0, *p* = 0.024) as a function of the pre-pulse dB intensity (F_4,102_ = 3.4, *p* = 0.011). This effect was mediated by phencyclidine and was not modified by nicotine. When the P was preceded by either the 70 dB pre-pulse (F_2,51_ = 3.4, *p* = 0.041) or the 80 dB pre-pulse (F_2,51_ = 6.4, *p* = 0.003), both groups exposed to phencyclidine (PCPacute and PCPNICacute) had reduced %PPI values when compared with the VEH group (Figure 5c).

## 4. Discussion

The epidemiological association between SCHZ and tobacco smoking is still poorly understood. Here, we used mice models to test the hypothesis that the sequence of developmental events associated with the establishment of these disorders impacts the comorbidity outcomes. Both locomotor sensitization and PPI, behaviors associated with SCHZ pathophysiology, were aggravated by nicotine exposure. Nicotine priming worsened phencyclidine-evoked hyperlocomotion. In contrast, PPI deficit aggravation was evident when SCHZ modeling preceded nicotine exposure. Distinctively, the assessment of rearing, a behavior not directly related to SCHZ symptoms, indicated that the developmental sequence of the comorbidity modeling was not determinant of the results. These data point to the importance of investigation of the initial stages of SCHZ and nicotine misuse comorbidity and imply that attention should be granted to the developmental timing of these disorders.

Hyperlocomotion and locomotor sensitization have been consistently identified in phencyclidine-exposed rodents. These features reflect neuroadaptations in the dopaminergic mesolimbic system [46,72,73] that mimic the dopaminergic alterations described in SCHZ patients [74,75,76] and have been associated with SCHZ-positive symptomatology [77,78,79]. Consistent with these findings, hyperlocomotion was identified from the first day of phencyclidine exposure onward and escalated during ACQ as a result of repeated exposures. These data corroborate previous studies on adolescent and adult rodents, indicating the development of locomotor sensitization [42,46,80,81].

Regarding nicotine, it may produce either depressant or stimulant effects on locomotor activity, depending on the age and dose [82,83,84]. Here, nicotine exposure caused a subtle hypolocomotor effect. Consistent with previous data, it faded with repeated exposures, which is indicative of development of tolerance [83]. Although the mechanisms that underlie the hypolocomotor effect of nicotine are still under investigation, there is evidence that they do not involve modulation of the dopaminergic [82] or serotonergic [85] neurotransmission, and that this drug’s effect on the endocannabinoid system might be involved [86]. Interestingly, previous studies further show that with repeated exposures, this period of nicotine-evoked hypolocomotion is followed by a hyperlocomotor effect [83,87]. However, a similar outcome was not identified in our study. This discrepant result could be explained by our choice of using adult mice, as adults are less susceptible than adolescents to the reinforcing effects of nicotine [84].

The analysis of our model of nicotine misuse and SCHZ comorbidity identified nicotine and phencyclidine interactions. However, the results varied as a function of the sequence of exposures. While nicotine exposure after modeling mice to SCHZ did not interfere with phencyclidine’s stimulatory effects, nicotine priming potentiated phencyclidine-evoked locomotor sensitization. Nicotine is known to modulate the activity of not only cholinergic and dopaminergic but also other groups of neurons, including the glutamatergic ones in the reward mesolimbic system [51,88]. Accordingly, neuroplastic events that occur during nicotine exposure and involve these systems most likely play a role in the nicotine-mediated potentiation of phencyclidine-evoked, psychotic-like behavior. The activity of ventral tegmental area dopaminergic neurons that project to the nucleus accumbens is indirectly modulated by nicotine binding to the nAChRs located in the terminals of glutamatergic neurons [51,88]. In addition, nicotine is known to upregulate subunits of both AMPA and NMDA receptors [89] and increase the AMPA/NMDA receptors’ current ratio of evoked EPSCs, an effect consistent with the increased synaptic plasticity mediated by the NMDA receptors that underlie the development of locomotor sensitization [90]. The aforementioned mechanisms most likely contribute to the development of locomotor sensitization identified in the SCHZ-modeled mice that were primed with nicotine. However, the question still remains for why a similar result was not present when nicotine exposure took place after SCHZ modeling. In this regard, blockage of the glutamate receptors through the administration of NMDA receptor antagonists reduces both nicotine-induced dopamine release in the nucleus accumbens and nicotine self-administration [89]. These effects suggest that a prior exposure to phencyclidine could result in subsequent impediment of the nicotine-mediated activation of NMDA receptors [89].

The PPI levels reflect adaptative sensorimotor gating processes. As a candidate endophenotype of SCHZ, it is reduced in SCHZ patients, in whom PPI impairment positively correlates with reduced global functioning measures and cognitive deficiency [57,58,91]. Here, we confirmed that phencyclidine effectively reduces PPI levels [42,92]. Repeated nicotine exposure after SCHZ modeling aggravated the PPI deficits, an effect restricted to males, which suggests that nicotine alters the course of SCHZ-related PPI deficits in a sex-selective manner. In contrast, both acute nicotine exposure and nicotine exposure prior to SCHZ modeling failed to improve or worsen the phencyclidine-evoked deficits. The lack of effect of acute nicotine exposure further suggests that nicotine interference on phencyclidine-mediated PPI deficits are dependent on events that occur in response to repeated exposures.

Previous studies reported that in the early stages of psychosis, PPI is reduced in tobacco smokers relative to non-smokers [93,94]. However, there is little information as to whether patients become smokers before or after SCHZ symptom expression, as well as evaluation of the contribution of these two possible sequences to the outcomes. Our results indicating that nicotine exposure per se does not interfere with PPI, and its deleterious effects were only identified after SCHZ modeling, suggesting that an a priori SCHZ-associated brain dysfunction needs to be present for nicotine to act upon. Accordingly, it is plausible that SCHZ-related neuroplastic events evoked by phencyclidine play a role in the nicotine-mediated aggravation of PPI deficits. In this regard, animal studies link PPI modulation to extended forebrain and pontine circuitry, and within this circuitry, manipulations such as NMDA receptor blockading and D2 receptor stimulation are known to be disruptive [58,93]. Even though the mechanisms that are responsible for nicotine’s effects on PPI are not established, there is clear evidence pointing to the involvement of α4β2 and α7 nAChRs [95,96], which in turn are known to modulate both the glutamatergic and dopaminergic systems [97]. Considering that phencyclidine was shown to be a negative allosteric modulator of nAChR [98], the nicotine-mediated worsening of PPI deficits in the mice primed with phencyclidine is consistent with a phencyclidine-mediated altered nAChR response to subsequent stimuli.

Sexual dimorphic differences are evident in several aspects of SCHZ, including prevalence, clinical presentation, and treatment efficacy [99]. For example, male SCHZ patients have more severe negative symptoms, while female patients express a more severe affective symptomatology [100]. Environmental factors are known to alter the developmental trajectories of neural circuits and, as a result, interfere with the disease’s process [101]. However, the sex-biased effects of environmental factors on the SCHZ symptomatology are still poorly explored. Here, punctual sex differences were identified at the end of the sensitization period, when females that were modeled to both SCHZ and nicotine misuse showed differential locomotor activities when compared with females only modeled to SCHZ. Even though we cannot rule out that those effects reflect an increased susceptibility of females to nicotine-mediated interference on SCHZ-like symptoms, their presence being restricted to the last day of the sensitization protocol, together with a lack of sex differences in the development of locomotor sensitization, render this unlikely. This latter possibility is consistent with evidence of a similar prevalence of SCHZ-positive symptoms in both genders [100] (but also consult [102,103]). It should be noted, however, that most studies did not consider the impact of smoking on the outcomes. Regarding PPI, we identified male-only increased susceptibility in response to co-modeling. Even though the underlying mechanisms are unclear, both the dopaminergic and glutamatergic systems show baseline sex differences in neurotransmitter concentrations, receptor expression, and synaptic responses [104,105]. In addition, it is known that nicotine affects these systems, and steroid hormones modulate nicotine’s effects through various mechanisms, including the binding and modification of nAChR function [105,106]. Previous findings point to an increased susceptibility of male SCHZ patients to PPI deficits. Even though there is little evidence that smoking contributes to this sex difference, smoking history data were not provided [107,108]. Therefore, future studies that investigate the sequence of events associated with SCHZ and nicotine misuse’s initial stages are warranted.

Rearing is an ethological behavior frequently used as a measure of exploration of a new environment. Even though it involves the activation of dopaminergic neurons in the mesolimbic system [109], rearing modulation occurs through mechanisms distinct from those that lead to locomotor hyperactivity [110,111,112]. Aside from this, distinct from both locomotor sensitization and PPI, there is scant evidence suggesting that rearing alteration models SCHZ symptoms or that this behavior is directly associated with nicotine dependence mechanisms. Accordingly, here, rearing was used as a means to infer whether nicotine and phencyclidine interactions specifically target SCHZ-like behaviors. We identified a robust inhibition of rearing in response to either nicotine or phencyclidine exposure, which corroborates previous findings [113,114,115]. Nicotine priming did not interfere with the phencyclidine-evoked effects on this exploratory behavior. Similarly, phencyclidine priming did not modify nicotine-mediated inhibition. In fact, nicotine’s effects were so predominant that phencyclidine priming followed by phencyclidine and nicotine’s combined exposure resulted in rearing inhibition that was similar to that identified in mice exposed only to nicotine. These data, together with our findings indicating the interference of nicotine in phencyclidine-evoked locomotor sensitization and PPI deficits, point to a mechanistic dissociation among behaviors and suggest the specific and limited interference of nicotine in phencyclidine-mediated SCHZ-like outcomes.

The limitations of the current study include the choice of using nicotine as a surrogate for tobacco smoking. Even though this neuroactive alkaloid is responsible for a wide variety of nervous system effects resulting from tobacco consumption [116], tobacco smoke contains about 4500 additional components, and there is evidence that tobacco compounds other than nicotine play important roles in tobacco’s effects on the central nervous system [117,118,119,120]. Accordingly, our experimental design did not rule out the possibility of functional interactions between other tobacco smoke components and phencyclidine in the regulation of SCHZ behavioral alterations. Another limitation is the choice of only one animal model of SCHZ. Even though the current data corroborate previous evidence that repeated phencyclidine exposure alters behaviors associated with SCHZ [57,121,122], future preclinical studies that use other SCHZ models could provide a more comprehensive profile of the impact of the sequence of events associated with the establishment of SCHZ and nicotine misuse on the comorbidity outcomes.

## 5. Conclusions

Tobacco smoking may begin either before or after SCHZ symptom manifestation. However, whether these two possible developmental sequences lead to distinct comorbidity outcomes is unknown. Here, we demonstrated that nicotine priming potentiated phencyclidine-evoked, SCHZ-like positive symptoms, while PPI aggravation was evident when SCHZ modeling preceded nicotine exposure. These data provide, for the first time, evidence that the sequence of events associated with SCHZ and nicotine misuse’s initial stages affects the comorbidity outcomes. Considering the established importance of environmental factors in SCHZ etiology and early pathophysiology events, future studies using animal models are warranted to further characterize the impact of the timing of nicotine exposure across the spectrum of SCHZ-like symptoms and the associated neurochemical alterations. Aside from this, attention should be granted to the sequence of development of these disorders in epidemiological studies. This knowledge has the potential to aid in the identification of mechanisms of nicotine interference in the neurobiology of SCHZ as well as facilitate the development of early intervention options aiming to ameliorate the prognosis of SCHZ.

## Figures and Tables

**Table 3:** Global analysis (mxANOVA) of PPI.

**Experiment 1: Effect or Interaction**	**F_d.f._, *p* Value**
dB Intensity	F_2,138_ = 40.7, *p* < 0.001
Phencyclidine	F_1,69_ = 29.8, *p* < 0.001
dB Intensity × Phencyclidine	F_2,138_ = 4.2, *p* = 0.017
dB Intensity × Phencyclidine × Nicotine × Sex	F_2,138_ = 2.6, *p* = 0.079
**Experiment 2: Effect or Interaction**	**F_d.f._, *p* Value**
dB Intensity	F_2,160_ = 61.5, *p* < 0.001
Phencyclidine	F_1,80_ = 7.6, *p* = 0.007
dB Intensity × Phencyclidine	F_2,160_ = 2.7, *p* = 0.07

## Data Availability

The raw data supporting the conclusions of this article are included in the article and Supplementary Materials. Further inquiries can be directed to the corresponding author.

## Supplementary Materials

The following supporting information can be downloaded at https://www.mdpi.com/article/10.3390/brainsci14090855/s1. Table S1: Summary of relevant papers with animal models of schizophrenia and nicotine exposure. Table S2: Body mass. Table S3: Global analysis (mxANOVA) of locomotor activity during the habituation phase. Table S4: Locomotor activity during the habituation phase (raw data file). Ref. [123] is cited in Supplementary Materials file.

## Abbreviations

ACQ = acquisition; AMPA = α-amino-3-hydroxy-5-methyl-4-isoxazolepropionic acid receptor; ENDS = electronic nicotine delivery systems; FPLSD = Fisher’s protected least significant difference; HAB = habituation; Int = interval; mANOVAs = multivariate ANOVAs; MK-801 = dizocilpin (antagonist of NMDA receptor); mxANOVAs = mixed-model analyses of variance; nAChRs = nicotinic acetylcholine receptors; NaCl = sodium chloride; NIC = nicotine; NMDA = N-methyl-D-aspartate receptor; OF = open field; P = pulse; PCP = phencyclidine; pP = pre-pulse followed by pulse; PPI = pre-pulse inhibition; s.c. = subcutaneous; SCHZ = schizophrenia; uANOVAs = univariate ANOVAs; VEH = vehicle.
