# Supplementary material for: Nicotine’s Effects on Schizophrenia-like Symptoms in a Mice Model: Time Matters
Source: Brain Sci. 2024 Aug 25;14(9):855. doi: 10.3390/brainsci14090855 (PMC11430416; doi:10.3390/brainsci14090855)
Supplement: Supplementary file 1 [file brainsci-14-00855-s001.zip › Dutra-Tavares et al 2024 SM S1 R1.pdf]

SUPPLEMENTARY MATERIAL S1

Nicotine effects on schizophrenia-like symptoms in a mice model: time matters

Ana Carolina Dutra-Tavares[1¶], Luciana Araújo Couto[2¶], Thainá P. Souza[2], Anais Bandeira-Martins[2], Juliana Oliveira Silva[2], Claudio C. Filgueiras[2], Anderson Ribeiro-Carvalho[3], Alex C. Manhães[2], Yael Abreu-Villaça[2*]

1. Departamento de Ciências Biomédicas e Saúde, Instituto de Biologia Roberto Alcantara Gomes, Universidade do Estado do Rio de Janeiro, Cabo Frio, RJ, Brazil.
2. Laboratório de Neurofisiologia, Departamento de Ciências Fisiológicas, Instituto de Biologia Roberto Alcantara Gomes, Universidade do Estado do Rio de Janeiro, Rio de Janeiro, RJ, Brazil.
3. Departamento de Ciências, Faculdade de Formação de Professores da Universidade do Estado do Rio de Janeiro, São Gonçalo, RJ, Brazil.

* Corresponding author: Dr. Alex Christian Manhães
Email: ac_manhaes@yahoo.com.br
ORCID: 0000-0003-4629-8343

[a] *In memoriam*

Table S1: Summary of relevant papers with animal models of schizophrenia and nicotine exposure

Authorship	Title	Model	Main findings
Peeters et al, 2024<br><br>doi: 10.1016/j.pbb.2024.173752	Modulation of mGlu5 reduces rewarding associative properties of nicotine via changes in mesolimbic plasticity: Relevance to comorbid cigarette smoking in psychosis.	Sprague-Dawley rats (male and female)<br><br>Quinpirole: 1.0mg/Kg, PN1-21<br><br>Nicotine: 0.6 mg/kg free base	Neonatal quinpirole treatment enhanced nicotine conditioned place preference.
Rodríguez-Veja et al, 2023<br><br>doi: 10.3390/ijms241914634	Nicotine exposure in a phencyclidine-induced mice model of schizophrenia: sex-selective medial prefrontal cortex protein markers of the combined insults in adolescent mice.	C57BL/6 mice (males and females)<br><br>Phencyclidine: 2.5mg/Kg, PN38-52; 10mg/Kg, PN53<br><br>Nicotine: 24mg/Kg/day, PN37-44	Proteins differentially expressed exclusively in co-exposed mice reveal unique effects associated with the comorbidity model. For females, proteins related to oxidative stress (peptidyl-prolyl cis-trans isomerase - Fkbp1a - and heat shock 70 kDa protein 1B - Hspa1b) and and energy metabolism subnetworks (gamma-enolase -Eno2), while in males a protein component of the synaptic transmission subnetwork (amphiphysin - Amph) were highlighted.
Wang et al, 2023<br><br>doi: 10.1038/s41401-022-00974-8	Nicotine pretreatment alleviates MK-801-induced behavioral and cognitive deficits in mice by regulating Pdlim5/CRTC1 in the PFC.	C57BL/6 mice (8- to 10-week-old, males only)<br><br>Nicotine: 0.2mg/Kg, for two weeks (Days 1-14)<br><br>MK-801: 0.2mg/Kg, i.p. (Day 15)	Pre-treatment with nicotine reversed MK-801-induced prepulse inhibition deficit and working memory impairment.
Dutra-Tavares et al, 2022<br><br>doi: 10.1016/j.taap.2022.116282	Adolescent nicotine potentiates the inhibitory effect of raclopride, a D2R antagonist, on phencyclidine-sensitized psychotic-like behavior in mice.	C57BL/6 mice (males and females)<br><br>Nicotine: 24mg/Kg/day, PN37-50<br><br>Phencyclidine: 10mg/Kg, PN45-50	Nicotine failed to interfere with phencyclidine stimulatory effects during acquisition phase (PN45-50).<br>During sensitization test (PN56), nicotine history shortened the expression of phencyclidine-evoked sensitization, however, nicotine challenge had no impact on locomotion.
Weeks et al, 2021<br><br>doi: 10.1093/ijnp/pyab064	Nicotine administration normalizes behavioral and neurophysiological perturbations in the MAM rodent model of schizophrenia.	Sprague-Dawley rats (adult)<br><br>Methylazoxymethanol acetate: 25mg/Kg, on gestational day 17<br><br>Nicotine: 0.3mg/Kg; s.c.<br>For 12-24days (duration of treatment between MAM and CTL rats being matched within experiments)	Chronic administration of NIC significantly improved deficits in prepulse inhibition of startle and novel object recognition among methylazoxymethanol acetate exposed rats.

Noda et al, 2021<br><br>doi:<br>10.1016/j.bbr.2021.113284	Multiple nicotinic acetylcholine receptor subtypes regulate social or cognitive behaviors in mice repeatedly administered phencyclidine.	Male ddY mice (six weeks old at the beginning)<br><br>Phencyclidine: PCP: 10 mg/kg/day, s.c. for 14 days (days 1-14)<br><br>Nicotine: 0.03 or 0.1 mg/kg/day, s.c. (Days 15-25)	Nicotine exposure attenuated phencyclidine-induced impairments in social or cognitive behaviors, decreased expression of α7 and/or α4 nAChR subunits in the prefrontal cortex; and increased expression of α7, α4, and β2 nAChR subunits in the nucleus accumbens.
Dutra-Tavares et al, 2021<br><br>doi:10.1371/journal.pone.025798	Does Nicotine Exposure during Adolescence Modify the Course of Schizophrenia-like Symptoms? Behavioral Analysis in a Phencyclidine-Induced Mice Model.	C57BL/6 mice (males and females)<br><br>Phencyclidine: 2.5mg/Kg, PN38-52; 10mg/Kg, PN53<br><br>Nicotine: 24mg/Kg/day, PN37-44	Nicotine history reduced the magnitude of phencyclidine-evoked hyperlocomotion and impeded the development of locomotor sensitization. It also mitigated the deficient sociability elicited by phencyclidine.
Brown et al, 2018<br><br>doi:<br>10.1159/000486391	Effects of Environmental Enrichment on Nicotine Sensitization in Rats Neonatally Treated with Quinpirole: Analyses of Glial Cell Line-Derived Neurotrophic Factor and Implications towards schizophrenia.	Rats<br><br>Quinpirole: 1.0mg/Kg, PN1-21<br><br>Nicotine: 0.5mg/Kg; PN38-45	Animals exposed to quinpirole presented nicotine sensitization
Waterhouse et al, 2016<br><br>doi:<br>10.1242/dmm.025072	Nicotine ameliorates schizophrenia-like cognitive deficits induced by maternal LPS exposure: A study in Rats.	Sprague Dawley rats (males only)<br><br>Maternal lipopolysaccharide: 0.5mg/Kg at gestation days, GD 10/11)<br><br>Nicotine: 0.6mg/Kg s.c. PN100-110	Repeated nicotine treatment led to improvements in performance of impaired prepulse inhibition and latent inhibition deficits caused by LPS model.
Scott and Taylor, 2014<br><br>doi:<br>10.1016/j.bbr.2013.11.009	Chronic nicotine attenuates phencyclidine-induced impulsivity in a mouse serial reaction time task.	C57BL/6 mice (males only)<br>Baseline 1:<br>Phencyclidine: 3mg/Kg, for two days<br>Baseline 2:<br>Nicotine: 200ug/mL, oral consumption for 14 days<br>Test session: nicotine (0.2mg/Kg, sc) or saline<br>Test sessions:<br>Phencyclidine: 3mg/Kg<br>Nicotine: 0.2mg/Kg, 30 minutes after phencyclidine exposure<br>Co-exposure for 10 days	No interaction between phencyclidine and nicotine was detected in attention.<br>Chronic oral, but not acute s.c. nicotine, attenuated phencyclidine-induced increase in impulsivity.
Berg et al, 2014<br><br>doi:<br>10.1111/adb.12082	Nicotine is more addictive, not more cognitively therapeutic in a neurodevelopmental model of schizophrenia produced by neonatal ventral hippocampal lesions.	Sprague-Dawley rats (male only)<br><br>Neonatal ventral hippocampal lesion as a model of schizophrenia (PN7)<br><br>Nicotine: 0.5 mg/kg, PN34-44 or 0.015mg/Kg, PN60 onwards	Nicotine exposure to adult rats did not reverse NVHL-induced cortical-hippocampal-dependent cognitive deficits.<br><br>Neonatal ventral hippocampal lesion increased adult nicotine self-administration, potentiating acquisition-intake, total nicotine consumed and drug seeking.
Berg et al, 2008<br><br>doi:<br>10.1016/j.neuropharm.2008.03.011	Accentuated behavioral sensitization to nicotine in the neonatal ventral hippocampal lesion model of schizophrenia.	Sprague-Dawley rats (male only)<br><br>Neonatal ventral hippocampal lesion as a model of schizophrenia (PN7)	Nicotine sensitization was greater in animals from neonatal ventral hippocampal lesion group and they also presented a greater locomotor response at

		Nicotine: 0.5 mg/kg,<br>For 3 weeks from PN60, followed by a challenge two weeks later	the challenge test.
Nespor and Tizabi, 2008<br><br>doi:10.1007/s00213-008-1220-x	Effects of nicotine on quinpirole- and dizocilpine (mk-801)-induced sensorimotor gating impairments in rats.	Sprague–Dawley rats (adult, female only)<br><br>MK-801:<br>0.15 mg/kg, i.p.<br><br>Quinpirole: 0.5 mg/kg, i.p.<br><br>Nicotine: 0.5-0.4mg/Kg, for the acute experiments; 0.2-0.4 for the chronic studies (daily for 1 week)	Administration of nicotine acutely or<br>chronically did not<br>have any effect on MK801-induced PPI impairment.<br><br>The higher dose of nicotine administered acutely significantly attenuated quinpirole-induced PPI impairment. Chronic daily administration of nicotine nearly normalized the quinpirole-induced impairments in PPI.
Rasmussen et al, 2008<br><br>doi:<br>10.1007/BF03033499	Effects of nicotine on sensorimotor gating impairment induced by long-term treatment with neurotoxic NMDA antagonism.	Sprague-Dawley rats (adult, female only)<br><br>NMDA antagonism neurotoxic regimen:<br>N-methyl-scopolamine (1 mg/ kg s.c.), then MK-801 (dizocilpine, 5 mg/kg i.p.) along with two separate doses (5 mg/kg) of pilocarpine<br><br>Nicoyine: 0.5 mg/kg, s.c., twice daily for 14 days, 10 days after the end of NMDA neurotoxic regimen (Day 10 – 23).	On day 17, PPI impairment was sustained in neurotoxically treated rats, regardless of saline or nicotine treatment. On day 22, however, the effect of neurotoxic treatment on PPI was totally absent in saline treated rats, whereas in nicotine treated rats, PPI impairment was still evident. Combination of nicotine and neurotoxic treatment also caused an up-regulation of high affinity nicotinic receptors in the cortex and the thalamus and apparent normalization of low affinity nicotinic receptors in the hippocampus.
Spielewoy and Markou, 2004<br><br>doi:<br>10.1023/B:BEGE.0000017878.75206.fd	Strain-specificity in nicotine attenuation of phencyclidine-induced disruption of prepulse inhibition in mice: relevance to smoking in schizophrenia patients.	C57BL/6J, DBA/2J, C3H/HeJ, and 129T2/SvEmsJ mice (8–10 weeks old, males only)<br><br>Phencyclidine: 10, 15 or 10mg/Kg, i.p.<br><br>Nicotine: 0.05, 0.50, or 1.0 mg/kg; 5 minutes after phencyclidine injection.	Nicotine attenuated the disruption in prepulse inhibition induced by phencyclidine in DBA/2J and C3H/HeJ but not in C57BL/6J or 129T2/SvEmsJ mice.
Tizabi et al, 1999<br><br>doi:<br>10.1007/s002130051078	Nicotine blocks quinpirole-induced behavior in rats: Psychiatric implications.	Rats (male and female)<br><br>Quinpirole: 1.0mg/Kg,<br>for the first 3 weeks from birth<br><br>Acutely during behavioral evaluation from PN23-29<br>Nicotine: 0.3mg/Kg i.p.<br>Quinpirole: 1.0mg/Kg	All effects of priming with quinpirole, namely, increased locomotor activity, paw treading, jumping and yawming, were partially or totally blocked with nicotine acute treatment.

Table S2 – Body Mass

**Experiment 1**					
	**Habituation (HAB)**				
	HAB1	HAB2	HAB3		
VEH	20.9±0.26	20.9±0.26	20.8±0.26		
	**Acquisition phase 1 (ACQ phase 1)**				
	ACQ1	ACQ2	ACQ3	ACQ4	
VEH	20.7±0.37	20.5±0.36	20.6±0.36	20.7±0.37	
PCP	20.7±0.48	20.7±0.35	20.7±0.35	20.8±0.35	
	**Acquisition phase 2 (ACQ phase 2) and prepulse inhibition (PPI)**				
	ACQ5	ACQ6	ACQ7	ACQ8	PPI
VEH	20.7±0.48	20.6±0.47	20.7±0.46	20.8±0.46	20.9±0.47
VEH/NIC	20.5±0.54	20.4±0.52	20.4±0.54	20.4±0.52	20.6±0.51
PCP/PCP	20.5±0.46	20.5±0.46	20.5±0.47	20.5±0.46	20.6±0.46
PCP/PCPNIC	21.0±0.53	20.9±0.52	20.9±0.52	21.0±0.51	21.1±0.51
**Experiment 2**					
	**Habituation (HAB)**				
	HAB1	HAB2	HAB3		
VEH	21.3±0.26	21.3±0.26	21.2±0.26		
	**Acquisition phase 1 (ACQ phase 1)**				
	ACQ1	ACQ2	ACQ3	ACQ4	
VEH	21.4±0.33	21.4±0.33	21.4±0.33	21.4±0.34	
NIC	21.0±0.37	20.9±0.36	20.9±0.36	20.7±0.36*	
	**Acquisition phase 2 (ACQ phase 2) and prepulse inhibition (PPI)**				
	ACQ5	ACQ6	ACQ7	ACQ8	PPI
VEH	21.3±0.45	21.3±0.44	21.4±0.44	21.4±0.44	21.6±0.44
VEH/PCP	21.3±0.53	21.2±0.50	21.2±0.51	21.2±0.53	21.3±0.53
NIC/NIC	20.5±0.60 †	20.5±0.61 †	20.4±0.61 †	20.4±0.61 †	20.5±0.61 †
NIC/PCPNIC	20.9±0.44	20.8±0.45	20.8±0.46	20.8±0.47	21.0±0.47

HAB1-HAB3: Days 1 to 3 of the Habituation phase; ACQ1-ACQ4: Days 1 to 4 of Acquisition phase 1; ACQ5-ACQ8: Days 1 to 4 of Acquisition phase 2; PPI: prepulse inhibition. * $p < 0.05$ *vs*. VEH group; † $p < 0.05$ *vs*. combined VEH + VEH/PCP groups.

Table S3 – Global analyses (mxANOVA) of locomotor activity during the Habituation phase

Experiment 1 - Effect or interaction	$F_{d.f.}$, p value
Day	$F_{2,178} = 89.2$, $p < 0.001$
Interval	$F_{1,89} = 708.9$, $p < 0.001$
Day × Interval	$F_{2,178} = 6.9$, $p = 0.001$
**Experiment 2 - Effect or interaction**	**$F_{d.f.}$, p value**
Day	$F_{2,158} = 131.9$, $p < 0.001$
Interval	$F_{1,79} = 1013.7$, $p < 0.001$
Phencyclidine × Sex	$F_{2,158} = 26.4$, $p < 0.001$

Table S4 – Locomotor activity during the Habituation phase (cm)

Experiment 1					
HAB1 (a)		HAB2 (b)		HAB3 (c)	
Int1	Int2	Int1	Int2	Int1	Int2
5278±119	3004±92***	4223±137[a]	2251±104***, [a]	4000±132[a, b]	2153±112***, [a]
**Experiment 2**					
HAB1		HAB2		HAB3	
Int1	Int2	Int1	Int2	Int1	Int2
5483±118	2893±92***	4035±122[a]	1969±84***, [a]	3604±113[a, c]	1920±103***, [a]

Values are means ± S.E.M. *** $p < 0.001$ pairwise comparisons between intervals 1 and 2 (Int1 and Int2); [a] $p < 0.001$ *vs*. Habituation (HAB) day 1 in a given interval; [b] $p < 0.05$, [c] $p < 0.001$ *vs*. HAB2 in a given interval.
